# A tailored compassion-focused therapy program for sexual minority young adults with depressive symotomatology: study protocol for a randomized controlled trial

**DOI:** 10.1186/s40359-017-0175-2

**Published:** 2017-03-01

**Authors:** Christopher A. Pepping, Anthony Lyons, Ruth McNair, James N. Kirby, Nicola Petrocchi, Paul Gilbert

**Affiliations:** 1 School of Psychology and Public Health, La Trobe University, Melbourne, Australia; 2 Australian Research Centre in Sex, Health, and Society, La Trobe University, Melbourne, Australia; 3 Department of General Practice, University of Melbourne, Melbourne, Australia; 4 School of Psychology, University of Queensland, Brisbane, Australia; 5 Department of Economics and Social Sciences, John Cabot University, Rome, Italy; 6 Department of Psychology, University of Derby, Derby, UK

**Keywords:** Lesbian, Gay, Bisexual, Depression, Compassion, Sexual minority

## Abstract

**Background:**

Lesbian, gay, and bisexual (LGB) men and women represent one of the highest-risk populations for depressive symptomatology and disorders, with young LGB adults being at greatest risk. To date, there have been no randomized controlled trials (RCT) to specifically target depressive symptoms in young LGB adults. This is despite research highlighting unique predictors of depressive symptomatology in this population. Here we outline a protocol for an RCT that will test the preliminary efficacy of a tailored compassion-focused therapy (CFT) intervention for young LGB adults compared with a self-directed cognitive behavioral therapy (CBT) program with no specific tailoring for LGB individuals.

**Methods:**

The CFT intervention consists of 8 units with self-directed reading and activities tailored to LGB young adults, and 8 x weekly 1-hour consultations with a therapist. The CBT intervention consists of 8 units with self-guided reading and activities, with 1 x 1-hour session with a therapist at the mid-point of therapy. Fifty LGB individuals with scores of 13 or above on the Beck Depression Inventory-II will be randomized to either the CFT or CBT condition. The primary outcome measure is depressive symptomatology. Secondary outcome measures are symptoms of anxiety, suicidal ideation, internalized homophobia, self-compassion, and shame and guilt proneness. Assessments will occur at pre-intervention, post-intervention, and at 3-month post-intervention.

**Discussion:**

This study is an RCT to test the preliminary efficacy of an LGB-tailored compassion-focused intervention for young LGB adults with depressive symptomatology. If this intervention is efficacious, this could begin to address the substantial mental health disparities amongst sexual minorities.

**Trial registration:**

ACTRN12616001018404. Prospective registration, registered 02/08/2016.

## Background

A large and coherent body of evidence reveals that LGB individuals experience poorer mental health than their heterosexual counterparts [1, 2]. Meta-analytic reviews indicate that gay men are between two and four times as likely to have attempted suicide, engaged in deliberate self-harm, or experienced depression, compared to their heterosexual counterparts [1]. Similarly, lesbian women are about twice as likely to have experienced depression or attempted suicide compared to heterosexual women [1]. Young LGB individuals are at greatest risk for depressive disorders. Specifically, LGB individuals in their teens and early 20s are more likely to suffer depression than both their heterosexual counterparts [3] and older LGB adults [4]. Suicide in this group is alarmingly high, with meta-analyses showing a 3-fold increase in the odds of suicidal ideation and attempted suicide in those aged younger than 21 years compared to their heterosexual counterparts [3].

These mental health disparities are largely accounted for by the impact of stigma, including internalized stigma or shame [5, 6]. Most LGB individuals report having experienced at least one incident of homophobic harassment, violence, or discrimination [6, 7]. Specifically, meta-analyses indicate that 44% of LGB individuals have been threatened with violence [8], 28% have been physically assaulted [9], and up to 80% have experienced verbal harassment [9]. In a large sample of heterosexuals and LGB individuals, perceived discrimination was related to lower quality of life, and increased indicators of psychiatric morbidity [6]. Importantly, when controlling for the effects of perceived discrimination, there were no differences in psychiatric morbidity between heterosexual and LGB individuals.

Although the risks associated with “coming out” have changed from previous generations for young LGB individuals [10], they still encounter significant life stressors such as family rejection [11] and discrimination [9]. Large numbers of LGB young people also face a range of developmental challenges related to their sexual orientation, such as coming to terms with their sexuality, “coming out” to family and friends, and experiences of discrimination and rejection. Importantly, the highest risk time for suicidal ideation and suicide attempts is when LGB individuals “come out” to their families [12]. To date, there has been no randomized controlled trial specifically designed to treat depression in LGB young adults. This is problematic, as there are unique issues that predict depressive symptomatology in LGB individuals and, in particular, LGB young adults, which interventions need to address. For instance, this population is exposed to a range of stigma-related or *minority stressors* [13], including *internalized* stigma and shame related to one’s sexual orientation, which predicts high risk for depression [14].

Many LGB people who seek help for mental health problems are not successful in getting the help they need [15]. Some report barriers such as a fear of discrimination [16] and lack of LGB sensitivity of services [17] and, of those who do access treatment, many report low satisfaction with treatment [15, 16]. These barriers can be partly overcome when using internet based support [17]. Recent research from our own team demonstrates that mental health interventions need to be tailored to be appropriate for LGB people, as existing interventions are often viewed as not appropriate, relevant, or inclusive of LGB individuals [18, 19]. Our team have also identified preferences that LGB young adults have regarding delivery and content of interventions to enhance efficacy [18, 19], and recently published the world’s first set of guidelines for tailoring therapy to the needs of LGB people [18].

One promising approach to helping LGB people with depression and depressive symtomatology is compassion-focused therapy (CFT). CFT is an evidence-based psychological intervention derived from a range of psychotherapies, and research in evolutionary, social and neuropsychology, along with the comtemplativre traditions [20]. This approach focuses on helping people access and cultivate care-focused motives and emotions to address issues of shame and self-criticism and build supportive inner resources [20]. Much evidence reveals that mindful compassion-based skills lead to a range of beneficial psychosocial outcomes, including increased self-esteem [21], more positive interpersonal relationships [22], and reduced symptoms of depression [23].

Since CFT was originally developed for and with individuals with high shame and self-criticism, it is likely to be particularly beneficial for LGB individuals for at least three reasons: (1) being compassionate predicts well-being in LGB individuals [24]; (2) internalized stigma is a significant predictor of depression in LGB individuals [14], and CFT directly reduces different types of shame and harsh self-criticism [25]; and (3) compassion-based interventions have been shown to be effective in reducing depressive symptoms (*d* = .86) [26], with a recent meta-analysis also reporting significant moderate effect sizes [27]. As mentioned, a significant predictor of mental health among LGB people is internalized stigma, or feelings of shame and low self-worth related to their sexual orientation [14]; indeed, the emphasis on “gay pride” in LGB communities is largely related to combatting and undermining internalized stigma [10]. Coupled with the non-judgemental awareness and mindful sensitivity to distress, CFT fosters sympathy, empathy, and distress engagement with a commitment to develop the wisdom and courage to alleviate and prevent distress. CFT uses concepts from evolutionary theory and research pertaining to the nature of sexuality and other motives and emotions and provides specific practices for emotion regulation, and strategies to switch from hostile self-criticism to compassionate self-support.

### Study objectives and hypotheses

The present RCT will test the preliminary efficacy of a newly developed tailored CFT intervention specifically designed to meet the needs of LGB young adults compared to a standard untailored CBT intervention. The aim is to assess whether a CFT intervention specifically tailored for LGB young adults will reduce depressive symptomatology and shame, and improve psychological functioning.

With regards to the primary outcome, it is hypothesized that, compared to the CBT condition, those in the CFT condition will demonstrate significantly lower symptoms of depression at post-intervention and 3-month follow-up compared to baseline pre-intervention levels. With regard to secondary outcomes, it is hypothesized that compared to those in the CBT condition, those in the CFT condition will demonstrate significantly lower symptoms of anxiety, suicidal ideation, internalized homophobia, and shame and guilt proneness, and significantly higher scores on a measure of self-compassion at post-intervention and 3-month follow-up compared to baseline levels.

## Method

### Participants and recruitment

Potential participants will respond to advertisements on social media for a free depression intervention for young LGB men and women aged 18–25 years. Recruitment will be nationwide around Australia, and advertisements will direct individuals to a brief survey. The survey will provide further information about the study as well as invite potential participants to provide their contact details and complete a brief screening questionnaire to assess their eligibility. Advertisements will be targeted to meet the following criteria for gay and bisexual male participants: Gender = male; romantic interest = male or male and female; aged 18–25; residing within Australia. Advertisements will be targeted to meet the following criteria for lesbian and bisexual female participants: Gender = female; romantic interest = female or female and male; aged 18–25; residing within Australia. The brief screening questionnaire consists of the Beck Depression Inventory-II [28], the Suicide Behaviour Questionnaire [29]; and key demographic information including age, gender, sexual orientation, ethnicity, whether participants are currently taking medication for depression, and whether they are currently engaged in another form of psychological therapy for their depression.

Participants will be included in the study if they meet the following inclusion criteria: (1) aged 18–25 years; (2) currently experiencing clinically significant depressive symptomatology, as evidenced by BDI-II scores of 13 and above; (3) and identify as gay, bisexual, lesbian, or non-heterosexual. Participants will be excluded from participating if they meet the following exclusion criteria: (1) currently receiving individual psychological intervention; (2) currently at *imminent* risk of harm to themselves, including planning or intending to engage in suicidal and/or para-suicidal behaviors. The presence of suicidal ideation is not an exclusion criteria. No other inclusion or exclusion criteria were used to assess eligibility for participation.

Following participants’ expressions of interest and completion of the screening questionnaire, a research assistant will assess eligibility based on the above criteria, and will contact potential participants to provide further details about the project. Specifically, the research assistant will outline that participants will be randomly assigned to one of two treatment conditions, answer questions, and provide potential participants with a consent form and the pre-assessment questionnaire. Ineligible participants will be referred to other services as appropriate.

### Study integrity, trial design, and procedure

The present study received ethical approval from the La Trobe University Human Research Ethics Committee (HEC016-10), and the trial was registered with the Australian New Zealand Clinical Trials Registry (ACTRN12616001018404). The present RCT adheres to the CONsolidated Standards Of Reporting Trials (CONSORT). The intervention protocol for both conditions will be manualized, and sessions with therapists for both conditions will be audio-recorded to enable quality assurance and assessment of protocol adherence.

The design will be a RCT involving 50 young adults, recruited over a five-month period (10 participants per month). This sample size was calculated using G*Power software to enable sufficient assessment of change over time in the study outcome measures. To assess change across three time points and between two conditions in a mixed-design ANOVA with both within (time) and between (condition) subjects effects, a sample of 42 is needed to detect a small-medium effect size of *f* = .20 at power = .80 and alpha = .05. Therefore, a sample of 50 will provide sufficient power while also accounting for possible attrition. Analyses will be conducted on an intention to treat basis.

Informed consent will be obtained prior to each participant commencing the study. Following initial screening described above, a research assistant will provide each participant with a pre-intervention questionnaire which will assess the primary and secondary outcome measures described below. Following completion of the pre-intervention questionnaire, participants will be randomly assigned to either the CFT or CBT condition with simple randomization using a computerized random number generator, and will be provided with the respective guidebooks. A therapist will contact participants approximately 1 week later to schedule session appointments. Participants will complete their respective intervention across an 8-week period. One-week post-intervention, a research assistant will provide each participant with a post-intervention questionnaire, which will again assess the primary and secondary outcome measures described below, as well as consumer satisfaction. Three-months post-intervention, the research assistant will provide participants with the follow-up questionnaire containing the primary and secondary outcome measures.

### Intervention procedures and delivery

Manualized treatment protocols have been developed for both the CFT and CBT interventions. Both interventions involve self-guided reading and activities, and some contact with a therapist via Skype, which will allow for people across the country to participate in the intervention. It will also test the intervention in a flexible delivery format to potentially enhance reach to LGB young adults who face barriers accessing face-to-face services [15, 16].

The CFT program is an 8-week compassionate mind training intervention (compassion-focused therapy) adapted to be appropriate for LGB young adults, and tailored to their unique life experiences. It consists of 8-units, each incorporating self-directed reading and experiential activities, coupled with weekly Skype sessions with a trained therapist to ensure compliance and to assist with tailoring skills to the specific individual’s situation. The eight units cover 1) psychoeducation pertaining to compassion, evolution, life challenges, brain functioning, and emotion systems to enhance understanding of the evolved functions that underpin emotions and behavior, and to facilitate de-shaming; 2) body-focused interventions (e.g., body posture, soothing rhythm breathing, voice tone exercises) to facilitate affiliative processing and the activation of the parasympathetic system, a core process in emotion regulation and mentalization (empathy/perspective taking); 3) cultivation of mindful attention, including non-judgmental awareness of experiences; 4) cultivation of the compassionate self as the persons motivational system through imagery practices. This also includes addressing the fears, blocks and resistances to compassion. Each of the units are tailored to cultivate the compassionate self, which is an orientation of the mind aimed at creating an inner secure base/safe haven for the individual to address and reduce shame and internalized stigma, and alleviate depression.

The units involve evidence-based step-by-step training founded on mindfulness and compassion-focused principles [20] and include examples relating to the lives of young LGB adults. For instance, included within the protocol is content pertaining to stigma and discrimination, significant life events such as coming out, and the effects of internalized homophobia and shame. These experiences of shame and internalized stigma are discussed in the context of negative societal attitudes to facilitate de-blaming and reduce shame. Specific exercises are included for participants to apply compassion-focused strategies to their own situation, with audio recordings of each exercise to facilitate practice between sessions.

Participants assigned to the CBT condition will receive a hard copy of an evidence-based cognitive-behavioral self-help book *Feeling Good: The New Mood Therapy* [30]. This cognitive behavioral bibliotherapy program guides the reader through a series of sections that provide psychoeducation about their symptoms of depression, and a series of self-directed cognitive and behavioral exercises to complete. This book has been established as beneficial for depression [31]. Participants will be provided with a guide to which sections of the book to read across the eight-week program. At the mid-point of this program (week 4), each participant will have a 1-hour telephone or Skype session with a therapist to monitor compliance, assist with any questions, and troubleshoot any difficulties with the exercises.

### Measures

#### Primary outcome

The primary outcomes will be depressive symptomatology, assessed by the Beck Depression Inventory-II (BDI-II) [28]. The BDI-II is a widely used 21-item self-report measure of depressive symptomatology which requires participants to respond to statements describing symptoms of depression on a scale from 0 (*never*) to 3 (*always*). The BDI-II specifies symptom severity from non-clinical to clinical ranges, and has demonstrated sound reliability and validity [32, 33].

#### Secondary outcomes

We also included several secondary outcome measures to assess whether the intervention is associated with a reduction in symptoms commonly associated with depression, including anxiety [34] and suicidal ideation [29]. In addition, we included secondary outcome measures associated with the targets of the CFT intervention described earlier, including internalized stigma [35], self-compassion [36] and shame [37].

The Beck Anxiety Inventory (BAI) [34] will be used to measure symptoms of anxiety. The BAI is a 21-item self-report measure of symptoms of anxiety, and demonstrates excellent validity, internal consistency, and test-retest reliability [34, 38]. The BAI asks participants to respond to a series of questions pertaining to symptoms of anxiety on a 4-point scale, ranging from 0 (*never*) to 3 (*always*).

The Suicide Behaviors Questionnaire-Revised [29] is a widely used 4-item self-report measure to assess suicidal ideation. The SBQ-R is a valid and reliable measure of suicidal ideation [29, 39], and effectively discriminates between non-suicidal and suicidal individuals [29].

The Lesbian, Gay, and Bisexual Identity Scale (LGBIS) [35] is a 27-item self-report measure that assesses eight minority stress-related constructs that each form eight subscales. Three subscales will be used in this research, which assess internalized stigma (Internalized Homonegativity subscale), motivations to conceal one’s sexual identity (Concealment Motivation subscale), and feeling positive about being LGB (Identity Affirmation subscale). The scale is measured on a 6-point Likert scale, ranging from 1 (*disagree strongly*) to 6 (*agree strongly*). The measure has demonstrable construct validity and internal consistency [35, 40].

The Self-Compassion Scale – Short Form [36] is a widely used 12-item measure of an individual’s capacity to experience feelings of kindness towards themselves, and to hold difficult feelings with warmth and concern as opposed to self-criticism. The 12 items are rated on a 5-point scale ranging from 1 (*almost never*) to 5 (*almost always*), and the measure has excellent psychometric properties, including validity and reliability [36].

The Guilt and Shame Proneness Scale [37] is a 16-item self-report measure of an individual’s feelings of shame and guilt. This will be included to assess shame and guilt proneness at a broad level, beyond sexual orientation. Participants respond to the items on a 7-point scale ranging from 0 (*unlikely*) to 4 (*very likely*). The measure is a valid and reliable measure of guilt and shame proneness, and demonstrates high test-retest reliability [37].

Finally, three measures to examine consumer satisfaction and acceptability will be included. The Consumer Satisfaction Questionnaire [41] is a widely used measure that assesses the helpfulness of an intervention or service. The LGBT Appropriateness Scale [18] is a 12-item measure of the extent to which LGB individuals perceive an intervention inclusive and relevant to them. Participants indicate whether they agree or disagree with the 12 statements pertaining to the suitability of the program for LGB individuals. Finally, participants in the CFT condition will rate the extent to which each of the eight core compassion-focused skills were helpful on a 5-point scale, ranging from 1 (*not at all helpful*) to 5 (*extremely helpful*).

### Statistical analyses

This RCT is a mixed-model repeated measures design involving between- and within-participants factors. The between-participants factor, or independent variable, is condition (CFT vs CBT). The within-participants factor is assessment time, with each participant assessed on relevant primary and secondary outcome measures at three time points (pre- and post-intervention, plus 3-month follow-up). A mixed-model repeated measures analysis of variance (ANOVA) will assess main and interaction effects across the three time points and between the two conditions. Participants in both conditions will also complete the BDI at each session with the therapist (sessions 1–8 for those in the CFT condition, at session 4 for those in the control). Should any participant not complete the post-questionnaire, their most recent BDI score will be used in order to maximize all available data. The effect size (Cohen’s *d*) and its precision will be calculated with 95% CI for the mean differences between pre-, post-, and follow-up for each condition.

## Discussion

LGB individuals experience poorer mental health than their heterosexual counterparts [1, 2], and young LGB individuals are at greatest risk for poor mental health, including depressive disorders [3, 4] and depression-related suicide [3]. Many LGB people who seek help for mental health problems are not successful in getting the treatment they need [15], and many face additional barriers to accessing effective treatment such as fears of discrimination about their sexual orientation. A recent RCT of a transdiagnostic, gay-affirmative intervention for gay and bisexual men aged 18–35 years (*M* age = 25.94) engaging in HIV-risk behavior was found to reduce symptoms of anxiety and depression [42], suggesting that tailored, gay-affirmative interventions hold considerable promise to enhance mental health. To date there has been no RCT to specifically target depressive symptomatology in LGB young adults. The present study therefore represents an important step toward addressing the disproportionate burden of depressive symptomatology in this population. In addition to the lack of research investigating interventions for LGB young adults, the extent to which current evidence-based interventions generalize to LGB individuals remains unclear. Specifically, the majority of studies invesitgating treatment for depression and depressive symptomatology do not report the sexual orientation of participants when describing their sample [43], which makes generalization to sexual minorities difficult. Finally, testing the intervention delivered via video-conferencing technology may have important clinical implications for enhancing reach to young LGB individuals who often face significant barriers to accessing mental health services.

It is important to note some limitations of the current study. First, although the inclusion of a self-directed active control condition is a clear strength of the current trial, it will not be possible to definitively establish whether any effects found are due to CFT, the LGB-tailoring, or to common factors such as therapist contact. Should the current CFT intervention show promise in the current trial, future randomized controlled trials should conduct a component analysis with a range of alternative control conditions to examine which specific components of the intervention are producing change. It is also important to note that there may be a range of potential moderators of treatment outcome, including social support and ‘outness’ to family and friends, and future research should examine whether these and other factors moderate outcome.

The present CFT intervention is designed to target the largest and well-established causes of depression in LGB individuals, such as shame and internalized homophobia. By doing so, we expect that the psychological impact of stigma will be reduced by this intervention. Nonetheless, it is important to note that there are some additional mediators that link stigma and discrimination with poor mental health. Specifically, a range of psychological, emotional, interpersonal, and behavioral processes have been established as mechanisms underlying the association between stigma and poor mental health [5]. Future researchers may therefore wish to explore the development and testing of additional interventions that target other specific mediators and to develop or refine interventions as new mediators are identified.

The present RCT will test the utility and preliminary efficacy of a newly developed CFT intervention specifically tailored to meet the needs of LGB young adults, compared to a standard CBT intervention that has not been tailored for LGB individuals. The aim is to assess whether a CFT intervention specifically targeted to address known causes of depression in LGB young adults will reduce depressive symptomatology and improve psychological functioning. Results of this research will have great potential to inform clinical practice and future research by examining the utility and preliminary efficacy of the first ever LGB affirmative treatment for depression in LGB young adults. This research will also help advance the fields of mindfulness, compassion, and LGB mental health by translating research into clinical practice, as well as delivering a much-needed intervention designed specifically to address a major mental health inequity.

## Abbreviations

CBT: Cognitive behavioral therapy
CFT: Compassion focused therapy
LGB: Lesbian, gay, and bisexual
